# Characterization of the complete chloroplast genome of *Syringa wolfii*

**DOI:** 10.1080/23802359.2020.1763216

**Published:** 2020-05-14

**Authors:** Jing Liu, Peipei Xiong, Wenpei Wang, Wenqi Wang, Wenting Zhang, Yan Qin, Jiuli Wang

**Affiliations:** a College of Ecological Environment and Resources, Qinghai Nationalities University, Xining, China; b Key Laboratory of Superior Forage Germplasm in the Qinghai-Tibetan Plateau, Qinghai Academy of Animal Science and Veterinary Medicine, Xining, China

**Keywords:** *Syringa wolfii*, complete chloroplast, genome, phylogenetic tree

## Abstract

*Syringa wolfii* (Syringa: Syringeae), an upright shrub, is ornamental species used in urban greenification. In this study, we determined the complete chloroplast (cp) genome of *S. wolfii* using next-generation sequencing (NGS). The size of the chloroplast genome is 156,571 bp in length, including a large single-copy region (LSC) of 86,684 bp, a small single-copy region (SSC) of 19,109 bp, and a pair of inverted repeat (IR) regions with 25,362 bp. The GC content of the chloroplast genome was 37.95%. Moreover, a total of 131 functional genes were annotated, including 87 protein-coding genes, 36 tRNA genes, and 8 rRNA genes. The neighbor-joining phylogenetic tree suggested that *S. wolfii* was closely related to *S. yunnansis*.

*Syringa wolfii* (Oleaceae: Syringeae), upright shrub, is an ornamental species used in urban greenification (Fu 1995). They mainly grow in hillside mixed wood forests, thickets, forest margins or riverside, or needle and broad-leaved mixed forests, at an altitude of 500–1600 m, which is mainly found in Heilongjiang, Jilin, Liaoning, China (Chen et al. 2007). *Syringa wolfii* has been used as medicinal plant in China because of its various medicinal components (Park et al. 1999). Recently, a series of chloroplast genome of *Oleaceae* were sequenced to conducted the phylogenetic relationships (Zhao et al. 2019). However, the phylogenetic location of *S. wolfii* has not been reported so far. Here, the complete chloroplast genome of *S. wolfii* (Genbank accession number: MN901631) was obtained based on high-throughput sequencing technology and the phylogenetic analysis of *S. wolfii* was carried out accordingly, which we expect will help future studies.

The samples of *S. wolfii* were collected from Xining Botanical Garden, Qinghai Province, China (36.62°N, 101.75°E). The experiment and analysis scheme are according to Wang et al. (2019). Total DNA of *S. wolfii* was extracted from the fresh leaves (about 3.0 g) with a modified CTAB method (Doyle and Doyle 1987). The voucher specimen (Specimen Accession number: WangJL2019201) was kept in the Herbarium of the Northwest Institute of Plateau Biology, Chinese Academy of Sciences (HNWP). Genome sequencing was performed using the Illumina HiSeq Platform (Illumina, San Diego, CA) at Genepioneer Biotechnologies Inc., Nanjing, China. Approximately 6.13 GB of clean data were yielded. The trimmed reads were mainly assembled by SPAdes (Bankevich et al. 2012) and SSPACE V2.0 (Marten et al. 2011). The assembled genome was annotated using CpGAVAS (Liu et al. 2012).

The complete chloroplast genome of *S. wolfii* is 156,517 bp in length with a typical quadripartite structure, containing a pair of inverted repeated (IR) regions of 25,362 bp, a large single-copy (LSC) region of 86,684 bp, and a small single-copy (SSC) region of 19,109 bp. The two IRs are separated by the LSC and the SSC. The GC content of the complete chloroplast genome was 37.95%. A total of 131 functional genes were annotated, including 8 rRNA genes, 36 tRNA genes, and 87 protein-coding genes. The rRNA genes, tRNA genes, and protein-coding genes account for 6.10%, 27.48%, and 66.41% of all annotated genes, respectively.

Phylogenetic relationships of *S. wolfii*, with 44 other species of Oleaceae, were resolved by means of the neighbor-joining method. Alignment was conducted using MAFFT (Katoh and Standley 2013; online version: https://mafft.cbrc.jp/alignment/server/). The neighbor-joining tree was built using MEGA 7 (Kumar et al. 2016) with bootstrap set to 1000. The neighbor-joining phylogenetic tree suggested that *S. wolfii* was closely related to *S. yunnansis* (Figure 1).

**Figure 1.**
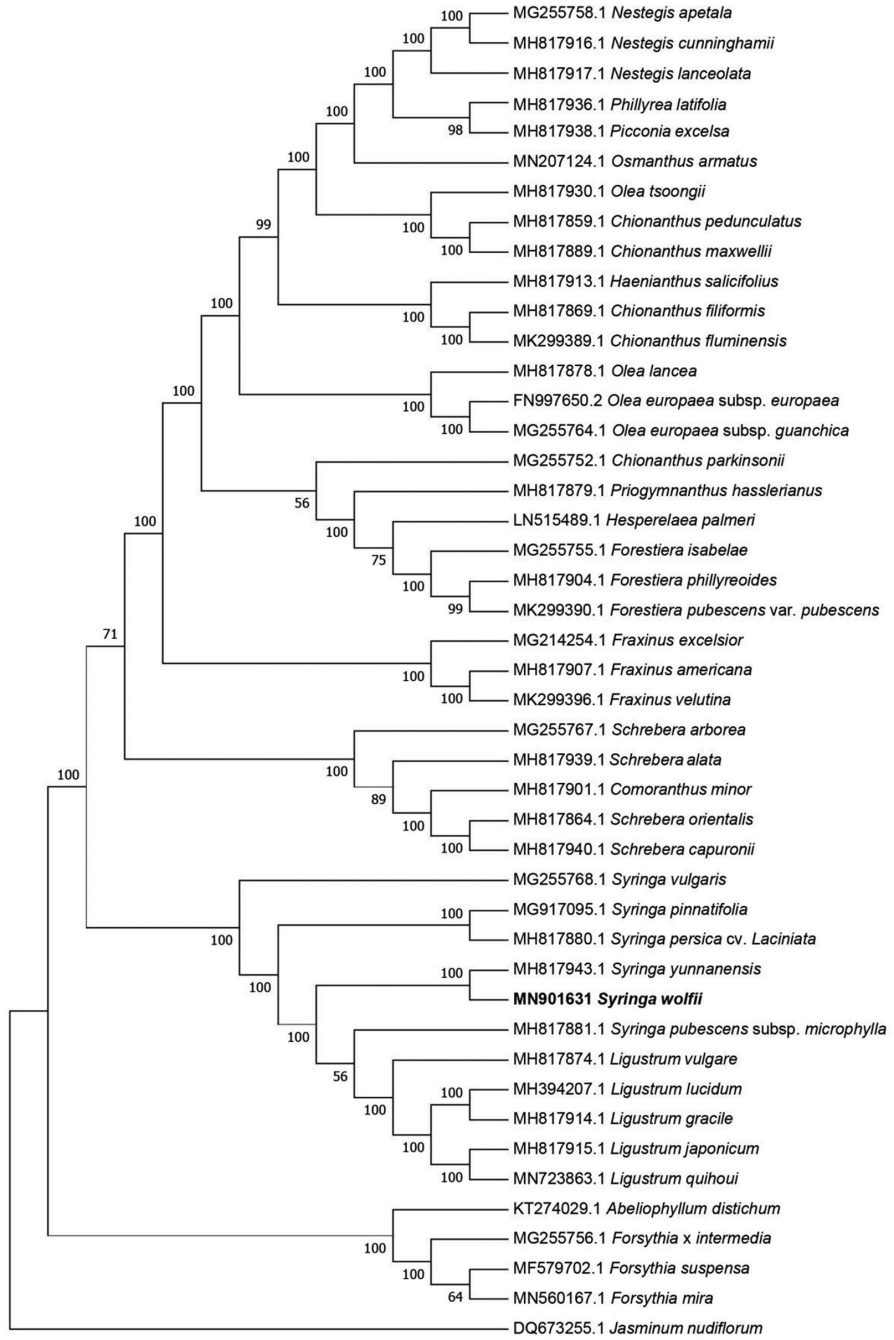
The neighbor-joining phylogenetic tree based on 45 chloroplast genome sequences.

## Data Availability

The data that support the findings of this study are openly available in Genbank at https://www.ncbi.nlm.nih.gov/genbank/, reference number MN901631.

## References

[CIT0001] Bankevich A, Nurk S, Antipov D, Gurevich AA, Dvorkin M, Kulikov AS, Lesin VM, Nikolenko SI, Pham S, Prjibelski AD, et al. 2012. SPAdes: a new genome assembly algorithm and its applications to single-cell sequencing. J Comput Biol. 19(5):455–477. 22506599 10.1089/cmb.2012.0021 PMC3342519

[CIT0002] Chen JY, Zhang ZS, Hong DY. 2007. A new status and typification of six names in *Syringa* (Oleaceae). Acta Phytotaxon Sin. 45(6):857–861.

[CIT0003] Doyle JJ, Doyle JL. 1987. A rapid DNA isolation procedure from small quantities of fresh leaf tissues. Phytochem Bull. 19:11–15.

[CIT0004] Fu P. 1995. Plant retrieval table for Northeast China. 2nd edn. Beijing: Science Press; p. 512.

[CIT0010] Katoh K, Standley DM. 2013. MAFFT multiple sequence alignment software version 7: improvements in performance and usability. Mol Biol Evol. 30:772–780. 23329690 10.1093/molbev/mst010 PMC3603318

[CIT0011] Kumar S, Stecher G, Tamura K. 2016. MEGA7: Molecular Evolutionary Genetics Analysis version 7.0 for bigger datasets. Mol Biol Evol. 33(7):1870–1874. 27004904 10.1093/molbev/msw054 PMC8210823

[CIT0005] Liu C, Shi L, Zhu Y, Chen H, Zhang J, Lin X, Guan X. 2012. CpGAVAS, an integrated web server for the annotation, visualization, analysis, and GenBank submission of completely sequenced chloroplast genome sequences. BMC Genomics. 13(1):715. 23256920 10.1186/1471-2164-13-715 PMC3543216

[CIT0006] Marten B, Henkel CV, Jansen HJ, Butler D, Pirovano W. 2011. Scaffolding pre-assembled contigs using SSPACE. Bioinformatics. 27(4):578–579. 21149342 10.1093/bioinformatics/btq683

[CIT0007] Park HJ, Lee MS, Lee KT, Sohn IC, Han YN, Miyamoto KI. 1999. Studies on constituents with cytotoxic activity from the stem bark of *Syringa velutina*. Chem Pharm Bull. 47(7):1029–1031. 10.1248/cpb.47.1029 10434406

[CIT0008] Wang J, Cao Q, Wang K, Xing R, Wang L, Zhou D. 2019. Characterization of the complete chloroplast genome of *Pterygocalyx volubilis* (Gentianaceae). Mitochondrial DNA Part B. 4(2):2579–2580. 33365634 10.1080/23802359.2019.1640644 PMC7706796

[CIT0009] Zhao YM, Yang ZY, Zhao YP, Li XL, Zhao ZX, Zhao GF. 2019. Chloroplast genome structural characteristics and phylogenetic relationships of Oleaceae. Chin Bull Bot. 54(4):441–454.

